# Species-specific characteristics of the biofilm generated in silicone tube: an in vitro study

**DOI:** 10.1186/s12886-018-0750-1

**Published:** 2018-04-03

**Authors:** Dong Ju Kim, Joo-Hee Park, Minwook Chang

**Affiliations:** Department of Ophthalmology, Dongguk University, Ilsan Hospital, 814, Siksadong, Ilsan-dong-gu, Goyang, Gyeonggido 410-773 South Korea

**Keywords:** Dacryocystorhinostomy, Biofilms, Silicone tube, Nasolacrimal duct obstruction, Pseudomonas, Staphylococcus

## Abstract

**Background:**

To investigate characteristics of biofilm which is usually found in silicone tube for nasolacrimal duct surgery and can be the root of chronic bacterial infections eventually resulted in surgical failure.

**Methods:**

To form a biofilm, sterile silicone tube was placed in culture media of *Staphylococcus aureus*, *Corynebacterium matruchotii*, *Pseudomonas aeruginosa*, or *Streptococcus pneumonia*. Biofilms formed on these silicone tubes were fixed with 95% ethanol and stained with 0.1% crystal violet. After staining, the optical densities of biofilms were measured using spectrophotometer on a weekly basis for 12 weeks.

**Results:**

*Staphylococcus aureus* group and *Pseudomonas aeruginosa* group formed significantly more amounts of biofilms compared to the control group. The maximum optical densities of the two groups were found on week 3–4 followed by a tendency of decrease afterwards. However, the amounts of biofilms formed in other groups of silicone tubes were not statistically significant from that of the control group.

**Conclusions:**

Bacterial species that could form biofilm on silicone tube included *Staphylococcus aureus* (week 3) and *Pseudomonas aeruginosa* (Week 4). It is important to first consider that the cause of infection around 1 month after silicone tube intubation can be *Staphylococcus aureus* and *Pseudomonas aeruginosa*.

## Background

Nasolacrimal duct obstruction (NLDO) mainly occurs in inflammation and fibrosis of lacrimal system. Either external or endoscopic dacryocystorhinostomy (DCR) is commonly used for NLDO [1–3]. DCR with silicone tube intubation has been commonly used to treat NLDO [4–8]. Although the beneficial effects of silicone tube intubation remain controversial [4, 9], silicone tube intubation is usually performed in order to maintain ostium patency and reinstate lacrimal drainage function, especially in case of distal or common canalicular obstruction [10, 11]. However, silicone tube intubation is associated with complications such as granulation formation, fibrosis and inflammation of nasolacrimal system, patient discomfort, infection of silicone tube, and cost related to intubation [12, 13]. Infection of silicone tube can result in postoperative failure [14, 15]. Bacteria can form biofilms, a complex of microbial communities enclosed in an exopolysaccharide matrix adherent to surface of prosthetics or living organism [16]. Biofilms enable bacteria to survive by reducing their metabolic needs and increasing their inherent resistance to antimicrobial agents. Biofilms formed on silicone tube could be the root of persistent and chronic bacterial infections. They can lead to chronic inflammatory response [17, 18]. Thus, it is important to find out the pathogen that formed biofilm on silicone tube.

In previous studies, both Gram-positive and Gram-negative bacteria have been isolated from extubated silicone tubes. Lee et al. [19] have reported culture positivity of 60% from extruded polyurethane nasolacrimal stents, with *Pseudomonas aeruginosa* being isolated from 40% of these stents. Ali et al. [20] have reported a positive culture of 94%, with *Pseudomonas aeruginosa* being isolated in 24% of cases. Kim et al. [14] have reported a positive culture of 94.9% from extubated silicone tubes, with 73% of the isolated bacteria being Gram-positive. They have also reported that *Pseudomonas aeruginosa* is associated with complications such as prolonged intubation, revision surgeries, and surgery failure [14].

The objective of this study was to investigate the characteristics of biofilms formed by four bacteria species (*Staphylococcus aureus, Corynebacterium matruchotii, Pseudomonas aeruginosa,* and *Streptococcus pneumonia*) usually found in silicone tubes used for nasolacrimal duct surgery [14, 19, 20]. The results of this study will improve our understanding on the characteristics of biofilms depending on bacteria, such as the amount of biofilms formed and the peak time of biofilm formation. These information will help us decide the treatment plan such as prophylactic use of antibiotics, the timing of stent removal and may aid in development of future strategies in treating silicone tube infection.

## Methods

### Bacteria culture

*Staphylococcus aureus* (KTCT#1621, ATCC#25923)*, Corynebacterium matruchotii* (KTCT#19325,) *Pseudomonas aeruginosa* (KTCT#2513, ATCC#9027), and *Streptococcus pneumonia* (KTCT#5765, ATCC#BAA-960) were used in this study. *Staphylococcus aureus, Corynebacterium matruchotii*, and *Streptococcus pneumonia* are Gram-positive bacteria while *Pseudomonas aeruginosa* is Gram-negative bacterium. All bacteria were obtained from the Korean Collection for Type cultures (KCTC). Bacteria were maintained in Nutrient broth media (234,000; BD)*,* BBL Trypticase soy broth media (211,768; BD), or Bacto Tryptic soy broth (211,825; BD), and cultured in an incubator at 37 °C except *Streptococcus pneumonia. Streptococcus pneumonia* was cultured in an incubator at 37 °C in an atmosphere of 5% CO_2_.

### Biofilm formation on silicon tube

Silicon tube (60–411-40; HelixMark) was cut into 2 cm in length and autoclaved. One silicone tube was cut into 6 pieces. Each group had 6 samples. To maintain the culture condition, we change the media as follows. *Staphylococcus aureus* culture media (Nutrient Broth media, 37 °C) was changed every 2 days. *Corynebacterium matruchotii* culture media (Trypticase soy broth, 37 °C) was changed every 4 days. *Pseudomonas aeruginosa* culture media (Trypticase soy broth, 37 °C) was changed every 2 days. *Streptococcus pneumonia* culture media (Bacto Tryptic soy broth, in 5% carbon dioxide at 37 °C) was changed every 4 days. The control group was not in contact with the bacteria in culture media (Nutrient Broth media).

### Biofilm formation measurement on silicon tube

**Fig. 1:**
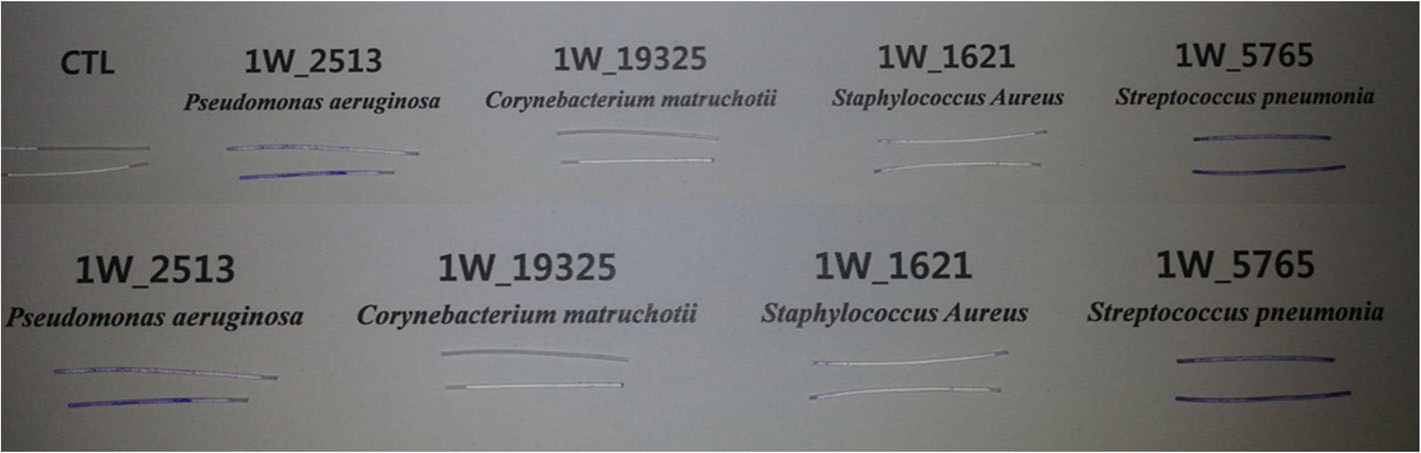
Sample of biofilm formed on silicone tube stained by crystal violet before measuring optical density by spectrophotometer

Silicon tubes incubated in cultured media were moved to new well and washed three times with distilled water. Biofilms formed on these silicon tubes were fixed with 95% ethanol. Tubes were washed twice with distilled water and stained with 0.1% crystal violet (V5265; SIGMA) for 30 min. After staining, silicon tubes were washed three times with distilled water. The crystal violet remained inside the silicon tube was removed using 22G syringe. These silicone tubes were dried on paper towel. The stained silicone tube was cut into 5 mm in thickness. These 5 mm tubes were placed in 96-well plate and filled with 95% ethanol (100 μl). The 96-well plate was sealed and incubated at 4 °C for 24 h. The optical density of the solubilized crystal violet in each well was then measured at wavelength of 570 nm using a spectrophotometer (SpectraMax plus 384 microplate reader, Molecular Devices, Sunnyvale, CA, USA). Each sample (Fig. 1) was measured for 12 weeks.

### Statistical analysis

The normality of data was checked by using Sapiro-Wilk test. All data showed normal distribution. The sphericity of the data was checked using Mauchly’s test. Repeated measure analysis of variance (RM-ANOVA) was used to compare time and optical density between control and bacteria. Post hoc test was conducted using the Bonferroni procedure. Statistical analyses were carried out using IBM SPSS ver. 21.0(IBM Corp., Armonk, NY, USA). *P*-value less than 0.05 was considered as statistically significant.

## Results

**Fig. 2:**
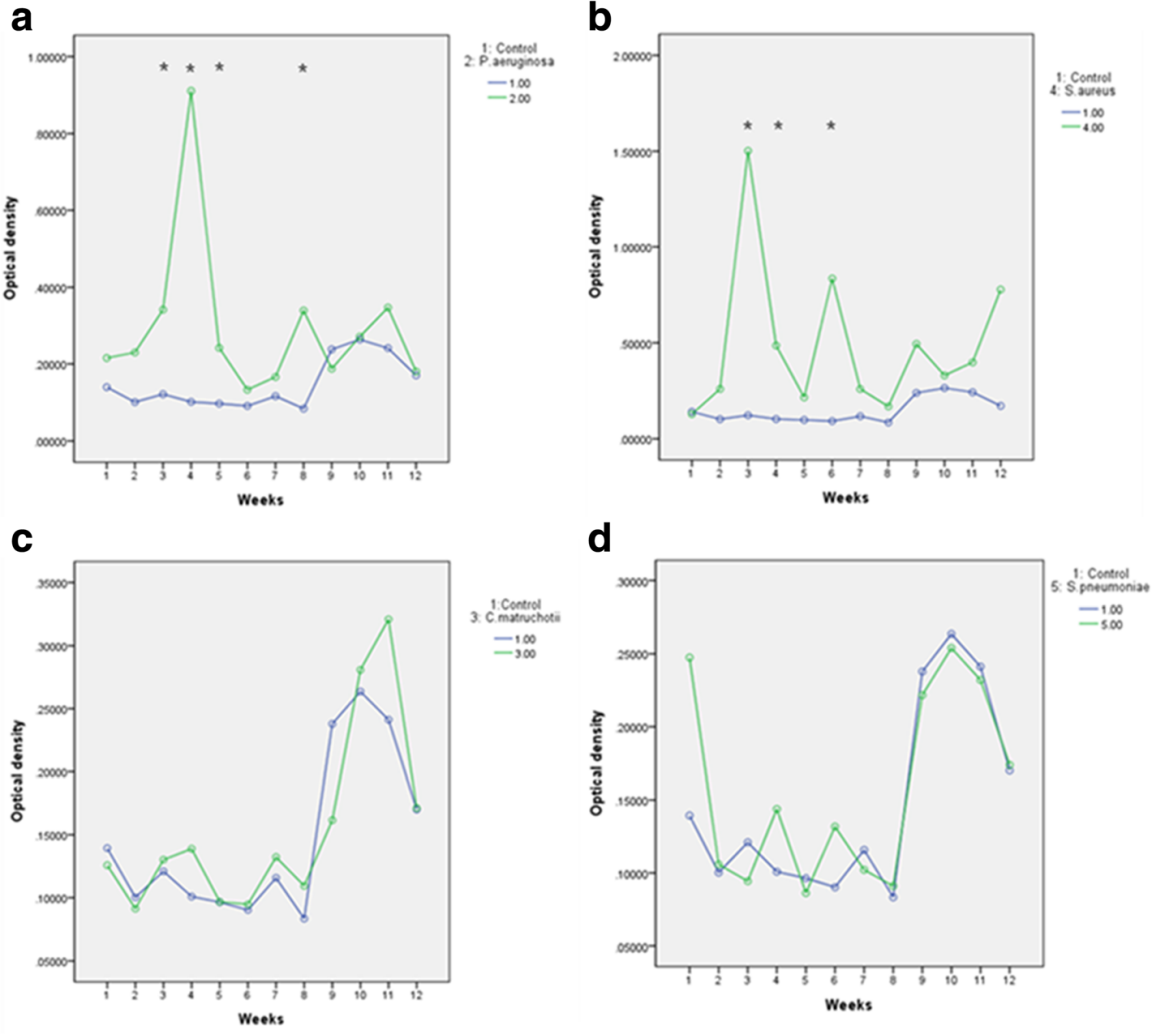
Overall tendency of optical density of biofilms formed according to time

Six tubes from each group were evaluated for 12 weeks. *Staphylococcus aureus* group (*P* = 0.000, RM-ANOVA) and *Pseudomonas aeruginosa* (*P* = 0.004, RM-ANOVA) group formed significantly higher optical density of biofilms compared to control groups. Specifically, significantly higher optical densities were observed at week 3, 4, 5, 8 in *Pseudomonas aeruginosa* group and at week 3, 4, 6 in *Staphylococcus aureus* group (Table 1, Fig. 2).

**Table 1:** Change of optical density according to time

	Optical density				
	Control	*P.aeruginosa*	*C.matruchotti*	*S.aureus*	*S.pneumoniae*
Week 1	0.1394 ± 0.0378	0.2154 ± 0.0740	0.1257 ± 0.0401	0.1272 ± 0.0406	0.2474 ± 0.1002
Week 2	0.1003 ± 0.0007	0.2299 ± 0.1030	0.0913 ± 0.0151	0.2583 ± 0.1249	0.1058 ± 0.0029
Week 3	0.1209 ± 0.0447	0.3414 ± 0.1359*	0.1301 ± 0.0459	1.5018 ± 0.2985*	0.0944 ± 0.0084
Week 4	0.1008 ± 0.0301	0.9106 ± 1.0651*	0.1388 ± 0.0517	0.4858 ± 0.1167*	0.1438 ± 0.0416
Week 5	0.0963 ± 0.0352	0.2415 ± 0.0691*	0.0967 ± 0.0228	0.2147 ± 0.0679	0.0863 ± 0.0074
Week 6	0.0902 ± 0.0028	0.1326 ± 0.1189	0.0946 ± 0.0034	0.8363 ± 0.2872*	0.1317 ± 0.0389
Week 7	0.1157 ± 0.0319	0.1659 ± 0.0416	0.1322 ± 0.0344	0.2579 ± 0.1053	0.1021 ± 0.0202
Week 8	0.0834 ± 0.0053	0.3392 ± 0.0757*	0.1094 ± 0.0332	0.1672 ± 0.0385	0.0911 ± 0.0222
Week 9	0.2378 ± 0.0257	0.1874 ± 0.0480	0.1616 ± 0.0410	0.4937 ± 0.1167	0.2217 ± 0.0179
Week 10	0.2635 ± 0.0613	0.2711 ± 0.0621	0.2808 ± 0.0388	0.3295 ± 0.0653	0.2539 ± 0.0357
Week 11	0.2412 ± 0.0403	0.3468 ± 0.0742	0.3209 ± 0.0746	0.3978 ± 0.0905	0.2320 ± 0.0035
Week 12	0.1701 ± 0.0461	0.1801 ± 0.0629	0.1712 ± 0.0519	0.7783 ± 0.2538	0.1738 ± 0.0210

Values are presented as mean ± standard deviation

*RM-ANOVA with post-hoc by Bonferroni (*P* < 0.004)

The maximum optical density of *Pseudomonas aeruginosa* was found in week 4. It was then decreased afterwards but increased temporarily in week 8. Similarly, the maximum optical density of *Staphylococcus aureus* was found in week 3 with a tendency to decrease afterwards. A second peak occurred in week 6. The amounts of biofilms formed by *Corynebacterium matruchotii* and *Streptococcus pneumonia* were not significantly (*P* > 0.05, RM-ANOVA) different from those of the control groups.

## Discussion

Biofilm have been associated with ocular prosthetic materials infection [21]. A biofilm is a complex organization of bacteria adherent to a biotic or an abiotic surface by living together in organized structures and communicating with one another in a co-operative manner [22, 23]. The bacteria self-produced polymeric matrix is embedded. This structure provides many advantages to bacteria, such as helping them endure environmental changes, resistant to host defense mechanisms, and resist conventional antibiotics. The presence of biofilm on a biomaterial could eventually lead to chronic inflammation and serve as a reservoir for bacteria. Therefore, bacterial biofilm has been increasingly recognized as playing an important role in surgical failure [24].

DCR and their surgical variants have been known as effective treatment for nasolacrimal duct problem. Among these surgical variants, nasolacrimal tube insertion is one of the most popular methods. However, complications related to postoperative infections associated with biofilms formed on tubes have been recently reported [12–15, 20].

Kim et al. [14] have reported that *Pseudomonas aeruginosa* infection is significantly associated with membranous obstruction of nasal mucosa, prolonged silicone intubation, and surgical failure. Balikoglu-Yilmaz et al. [25] have reported that *Staphylococcus epidermidis* and *Pseudomonas aeruginosa* are commonly culture positive on lacrimal stent. Ali et al. [20] have also reported that the most common bacterial organisms on lacrimal stents are *Pseudomonas aeruginosa* and *Staphylococcus aureus.* However, biofilm may be formed even if the culture was negative for bacterial growth. [26] due to limitation of conventional culture techniques [27]. Therefore, the possibility of chronic infection caused by biofilm could not be ruled out when the culture was negative.

Bacteriology of dacyrocystitis has been gradually changed. Imatiaz A. Chaudhry et al. and Hartikainen J et al. have reported that *Staphylococcus* species were usually the most common organisms in Gram-positive bacteria while *Pseudomonas aeruginosa* and *Haemophilus* species were common Gram-negative bacteria found in dacyrocystitis. Corynebacterium species were also detected [28–30]. Also, *Pseudomonas aeruginosa* and *Staphylococcus aureus* are well known producers of biofilms in paranasal sinus disease [31]. However, according to studies by David B. Samimi et al., nontuberculosis mycobacteria(NTM) was detected in silicone tube. Particularly, NTM was found in patients with clinically significant infection of silicone tube. But this study was a single institutional study in South Florida, so it is difficult to apply it to other region [32, 33]. Considering all above studies, we selected four bacterial species in this study, including *Staphylococcus aureus* and *Pseudomonas aeruginosa*.

The optical densities of the *Staphylococcus aureus* group and the *Pseudomonas aeruginosa* group were found to be higher than those of the control groups. This result suggests that the formation of biofilm depends on bacteria species. In terms of the amount of biofilm formed, the maximum value was achieved in 3 or 4 weeks with a tendency of decrease afterwards. A secondary peak occurred at 3 or 4 weeks after the first peak. This result can be used as a basis to use prophylactic antibiotics for 4 weeks to 8 weeks after silicone tube intubation. Once infection occurs, to treat infection and prevent recurrences, the prosthetic medical devices must be removed and antibiotics must be used at stronger doses or more often. It has been reported that if lacrimal stents are left longer than 1 month, biofilms may influence postoperative healing and the ultimate outcome [34]. Our results also support the use of prophylactic antibiotics after surgery.

This study has some limitations. First, this is an in-vitro study. Therefore, interaction of bacteria and immune system could not be evaluated. The causative relationship between biofilm and surgical failure was difficult to determine. To evaluate this, an in-vivo study is required. Second, many studies have reported that both bacteria and fungus are isolated from silicone stents, with fungus being isolated from 3.8% to 60% of cases [14, 20, 26, 34]. Symbiotic biofilms are more resistant to antibiotics with more complicated complex compared to non-symbiotic biofilms [35, 36]. However, we only investigated bacteria in this study.

Third, there are many methods for quantifying and detecting biofilms such as scanning electron microscopy morphology as a predictor [32, 37], biomass using confocal scanning laser microscope [34]. However, there is no standard method. We selected optical density using spectrophotometer at wavelength of 570 nm. This method had limited ability in assessing the depth of biofilm, thickness, or maturity. However, it can be quantified for comparison purpose and one study reported that crystal violet detect biofilm matrix for monitoring overall biofilm architecture [38, 39]. Antibiotic sensitivity or resistance associated with optical density could provide better information on treatment decision.

## Conclusions

In conclusion, this study found that, of four bacterial species tested, *Staphylococcus aureus* and *Pseudomonas aeruginosa* could significantly form biofilms on silicone tube. The maximum optical density of biofilms occurred at around 1 month after incubating silicone tubes with bacterial culture media. A secondary peak occurred at around 2 months after incubation. On this basis, we first consider that the cause of infection around 1 month after silicone tube intubation can be *Staphylococcus aureus* and *Pseudomonas aeruginosa*.

## Abbreviations

DCR: dacryocystorhinostomy
NLDO: nasolacrimal duct obstruction
NTM: nontuberculosis mycobacteria
OD: optical density
